# Start-Up Circuits for Ultra-Low-Voltage Thermoelectric Energy Harvesting: A Topology-Oriented Review and Design Guide

**DOI:** 10.3390/nano16100586

**Published:** 2026-05-11

**Authors:** Muhammad Ali, S. Jarjees Ul Hassan, Sungbo Cho

**Affiliations:** 1 Department of Semiconductor Engineering, Gachon University, Seongnam-si 13120, Republic of Korea; 2 Centre for Energy Technologies, Department of Business Development and Technology, Aarhus University, Birk Centerpark 15, 7400 Herning, Denmark; 3 Department of Electronic Engineering, Gachon University, Seongnam-si 13120, Republic of Korea; 4 Department of Health Sciences and Technology GAIHST, Gachon University, Incheon 21999, Republic of Korea

**Keywords:** energy harvesting, TEG, start-up, boost converter, ring oscillator, self-power systems

## Abstract

Thermoelectric generator (TEG)-based energy harvesting (EH) has emerged as a promising solution for powering ultra-low-power electronic systems. However, the inherently low output voltage of miniature TEGs is often below a range of 40–100 mV under small temperature gradients, presenting a fundamental cold-start challenge for DC-DC boost converters, preventing fully autonomous operation without dedicated start-up circuitry. Although numerous start-up techniques have been reported, the existing literature lacks a focused, design-oriented review of circuit architecture specifically optimized for ultra-low-voltage TEG applications. This paper addresses this gap by introducing a unified classification framework and providing a structured, topology-oriented analysis of state-of-the-art start-up strategies for TEG-based EH systems. Reported techniques are organized into five categories: external energy assistance, mechanical switch-assisted techniques, multi-source EH, transformer-based architectures, and oscillator-driven DC-AC-DC conversion. Each category is comparatively evaluated in terms of start-up voltage, integration level, efficiency, and system autonomy. Among these, oscillator-based approaches, particularly ring oscillator (RO) architectures, emerge as the most viable pathway toward fully integrated and scalable implementations, owing to their CMOS compatibility and architectural flexibility. The review further discusses key design trade-offs, handover stability challenges, and practical limitations, and provides architectural insights to guide the development of next-generation autonomous TEG-powered platforms.

## 1. Introduction

Energy harvesting (EH) refers to the process of capturing and converting ambient energy from surrounding environmental sources, such as light (photovoltaics) [1,2], thermal gradients (thermoelectric generators) [3,4], mechanical vibrations (piezoelectric, triboelectric nanogenerators) [5,6,7], and radiofrequency (RF) waves [8,9], into electrical power. By enabling battery-less or battery-assisted operation, EH has emerged as a key enabler for ultra-low-power electronic systems, offering a sustainable alternative to conventional batteries that are inherently limited by finite lifetime, size constraints, and maintenance requirements [10,11,12]. In parallel, electrochemical energy-conversion systems, including urea electrooxidation, methanol electrooxidation, direct methanol fuel cells, and biomass have also attracted increasing attention as sustainable routes for efficient energy conversion and portable power generation [13,14,15,16]. These advantages are particularly critical for applications such as biomedical implants [17], wearable electronics [18], and Internet-of-Things (IoT) sensor nodes [19], many of which are deployed in remote or inaccessible locations where battery replacement is impractical. As the number of connected devices continues to grow rapidly, the development of scalable, maintenance-free, and environmentally sustainable power solutions has become increasingly urgent [20,21].

In most energy harvesting systems, the power-management circuit must communicate with a low, variable, and frequently high-impedance energy source [11]. Depending on the source, this interface may include rectifiers for AC harvesters, impedance-matching networks for RF and piezoelectric harvesters, maximum power point tracking circuits for photovoltaic and thermoelectric sources [22,23], DC-DC converters, energy-storage elements, protection circuits, and output voltage regulators [16,19]. Despite significant advances in these circuits, several common challenges remain, including low input power, time-varying source conditions, impedance mismatch, high control-circuit overhead, reduced conversion efficiency at microwatt or nanowatt power levels, and the difficulty of achieving fully integrated implementation [6,24,25,26,27,28]. Among these problems, cold-start functionality is especially important since the power-management circuit requires a minimum supply voltage to commence normal functioning. This issue is especially acute in TEG-based energy harvesting, where the generated voltage under tiny temperature gradients is frequently only a few tens of millivolts.

**Figure 1:**
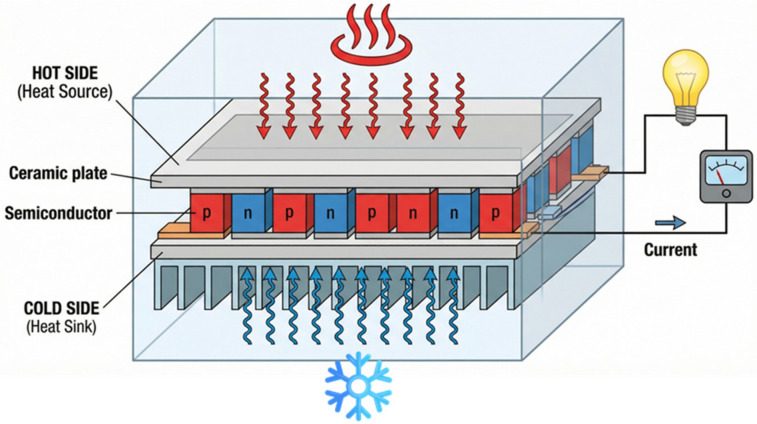
The structure of a thermoelectric generator.

Among the various ambient sources, thermal energy harvesting using thermoelectric generators (TEGs) is especially attractive due to the widespread availability of waste heat in industrial processes, automotive systems, and even biological environments [24,25,26]. TEGs operate as solid-state devices with no moving parts, providing high reliability and continuous energy generation whenever a temperature gradient is present [27,28]. Their operation is primarily governed by the Seebeck effect, whereby an electromotive force is generated across a material subjected to a temperature difference (∆T) [29]. Figure 1 illustrates that by electrically connecting p-type and n-type thermoelectric elements in series, a usable output voltage (*V*_OUT_) proportional to the temperature gradient (∆T) can be obtained, *V*_OC_ = α × ∆T where V_OC_ is the open-circuit voltage and α is the Seebeck coefficient.

Despite ongoing advances in thermoelectric materials [30], the practical deployment of TEG-based EH systems remain fundamentally constrained by the low *V*_OUT_ they generate under modest temperature gradients (∆T). In many realistic scenarios, the open-circuit voltage of miniature TEGs is limited to only tens or a few hundred millivolts [31,32,33,34,35,36], which is far below the minimum operating voltage required by conventional CMOS circuits [33,34]. This voltage mismatch presents a major power management challenge, as it prevents the direct activation of standard electronic systems. To bridge this gap, a DC-DC boost converter is required to raise the harvested voltage to a usable level [32,35,37]. However, the boost converter itself requires a minimum supply voltage to initiate operation, leading to a well-known “cold-start” dilemma [38,39].

**Figure 2:**
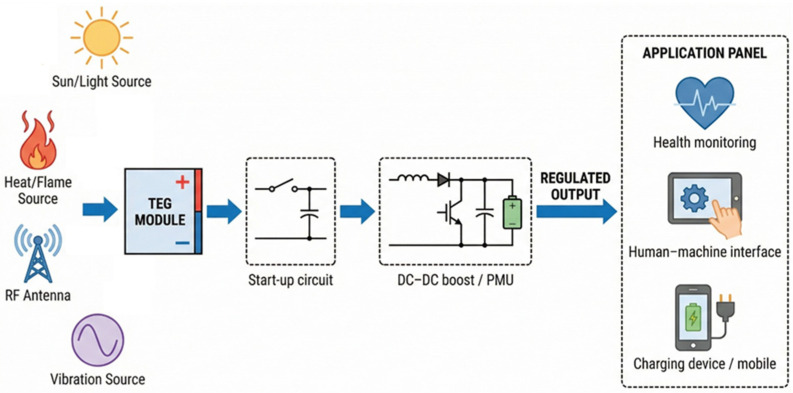
Complete TEG-based energy harvesting power management unit architecture, showing the cold-start circuit, main boost converter, energy-storage element, output regulation stage, and regulated power supply for multiple application loads.

Figure 2 illustrates the block-level architecture of a TEG-based energy harvesting power management unit and places the start-up circuit within the complete PMU signal chain. During cold start, the TEG output voltage, *V*_OUT_, is insufficient to power the control circuitry of the main boost converter [40,41]. Therefore, a dedicated start-up circuit is required to accumulate energy from the ultra-low-voltage source and initiate the voltage-conversion process [35]. After the internal supply voltage reaches the required level, the main DC-DC boost converter takes over and transfers energy to the storage element. The complete PMU also requires auxiliary functional blocks, including an input/MPPT interface, voltage/reference generation circuits, control logic, protection and undervoltage-lockout circuits, and an output regulation/load-management stage. These output circuits are necessary to provide a stable regulated voltage to downstream loads such as IoT nodes, biomedical sensors, and portable electronics [17,37,42,43]. As a result, the start-up circuit is a critical but not standalone component of a complete TEG power-management system [44].

Over the past two decades, a wide range of start-up circuit topologies has been proposed to address this challenge, including battery-assisted approaches [45,46], transformer-based architectures [47,48], multi-source energy harvesting schemes [10,49], and fully integrated self-oscillating solutions [50,51,52]. While several review papers have discussed EH systems and power management units at a high level [50,53,54], a focused, topology-oriented analysis of start-up circuits specifically targeting sub-100 mV TEG operation remains limited. This gap is significant, as different start-up strategies exhibit fundamentally different trade-offs in terms of achievable start-up voltage, integration level, efficiency, and system complexity [55,56].

To address this need, this paper presents a design-oriented architectural review of ultra-low-voltage start-up circuits for TEG-based EH systems. Reported approaches are unified within a consistent classification framework and comparatively analyzed based on key performance metrics and practical design constraints [57,58,59]. By synthesizing recent advances and highlighting unresolved challenges, this review aims to provide a practical reference for circuit designers developing next-generation autonomous EH platforms.

Unlike prior reviews that focus broadly on energy harvesting systems or power management units, this work provides a design-oriented architectural perspective specifically targeting ultra-low-voltage start-up circuits for thermoelectric energy harvesting. By unifying disparate approaches under a consistent classification framework and emphasizing the feasibility of integration, autonomy, and circuit-level trade-offs, this review provides practical guidance for selecting start-up architectures in emerging self-powered systems.

The remainder of this paper is organized as follows. Section 2 reviews and classifies existing start-up circuit topologies based on their operating principles. Section 3 presents a comparative analysis and a topology selection guide to assist design decisions under ultra-low-voltage constraints. Section 4 discusses fundamental limitations, including subthreshold operation constraints, and outlines future research directions. Finally, Section 5 concludes the paper.

## 2. Start-Up Circuits Techniques

In typical EH applications, the system often begins in a fully depleted state, necessitating a dedicated cold-start block to initiate operation without relying on pre-charged external storage elements such as batteries or capacitors [60,61,62]. This start-up block must bootstrap the system using only TEG’s inherently low and often insufficient *V*_IN_ (TEG terminal voltage). The primary design objective for this circuit is to minimize the start-up voltage (*V*_start_), as this metric directly determines the minimum temperature gradient from which the system can autonomously begin harvesting [63,64]. Consequently, secondary parameters such as power conversion efficiency and charging rate are often traded off to achieve the lowest possible *V*_start_.

**Figure 3:**
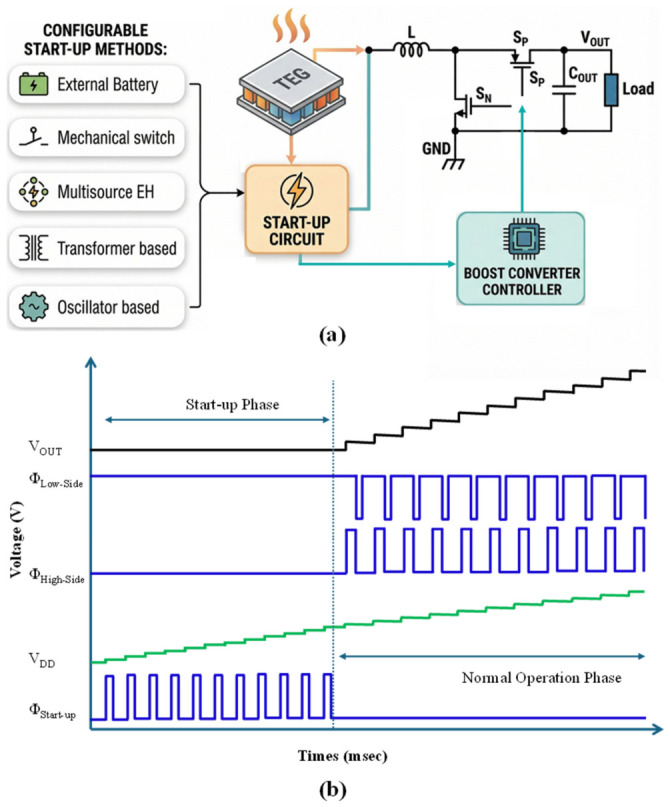
(**a**) Block diagram of start-up approaches for the TEG boost converter. (**b**) Waveform illustration of start-up and normal operation phases.

This operating paradigm is illustrated in Figure 3, which depicts a standard boost converter and its accompanying start-up circuit block. The design of this start-up block is the core differentiator among reported approaches, which can be broadly classified into external assistance (e.g., battery) [65], passive component-assisted techniques (e.g., mechanical switch, transformer) [66,67], multi-source harvesting [68,69], and fully integrated active circuits (e.g., oscillator-driven) [70,71]. Such converters typically require an initial internal supply of ≈500 mV (or higher, depending on process and controller design) to power their control circuitry before normal switching operation can begin. An auxiliary start-up circuit provides this critical initial energy pulse. The system-level benefit is significant: since the main converter can often operate steadily from input well below 100 mV, a low *V*_start_ circuit enables harvesting from minimal thermal gradients [72,73]. Once the system is successfully bootstrapped, the start-up block is disabled to eliminate quiescent power drain, thereby maximizing steady-state efficiency.

Figure 3b illustrates the operational waveform during the start-up and normal operation phases. During the start-up, the TEG’s input voltage (*V*_IN_) activates the start-up circuit, which generates a pulse train (Φ_Start-up_). During the high phase of Φ_Start-up_, the *V*_IN_ charges the boost converter inductor (*L*). During the low phase of ΦStart-up, the energy stored in *L* is transferred to the internal supply reservoir capacitor (*C*_DD_), which progressively raises the internal supply voltage (*V*_DD_) until the main converter becomes operational.

For a pulse width *t*_on_, the inductor current during the high phase can be approximated as

(1) $$I_{L}\left(t_{on}\right)=\frac{V_{\mathrm{IN}}}{L}\times t_{on}$$

The energy stored in the inductor is

(2) $$E_{L}=\frac{1}{2}LI_{L}^{2}$$

(3) $$E_{L}=\frac{1}{2}L{\left(\frac{V_{IN}}{L}t_{on}\right)}^{2}=\frac{V_{IN}^{2}t_{on}^{2}}{2L}$$

Assuming this energy is delivered to the reservoir *C*_DD_, the incremental rise in *V*_DD_ can be expressed as

(4) $$∆V_{DD}=\frac{E_{L}}{C_{DD}V_{DD}}\approx \frac{V_{IN}^{2}t_{on}^{2}}{{2LC}_{DD}V_{DD}}$$

With repeated pulses, *V*_DD_ ramps upward until it crosses the controller’s enable threshold. The system then transitions to normal operation, where the controller generates complementary gate signals for the high-side (Φ_High-side_) and low-side (Φ_Low-side_) switches. Under regulated switching, the output voltage (*V*_OUT_) rises toward its steady-state target, completing autonomous initialization.

### 2.1. External Battery Assistance

A foundational, though ultimately limiting, start-up strategy involves the use of an external energy source, such as a battery [74,75] or a pre-charged capacitor [76]. While conceptually straightforward, this method introduces significant system-level drawbacks. It necessitates diverting a portion of harvested energy to replenish the auxiliary storage for subsequent start-up cycles, reducing the net energy delivered to the load. More critically, if the storage element is fully depleted, the system cannot self-recover, thereby violating energy autonomy, an unacceptable condition for remote sensing and biomedical implants where maintenance is impractical.

**Figure 4:**
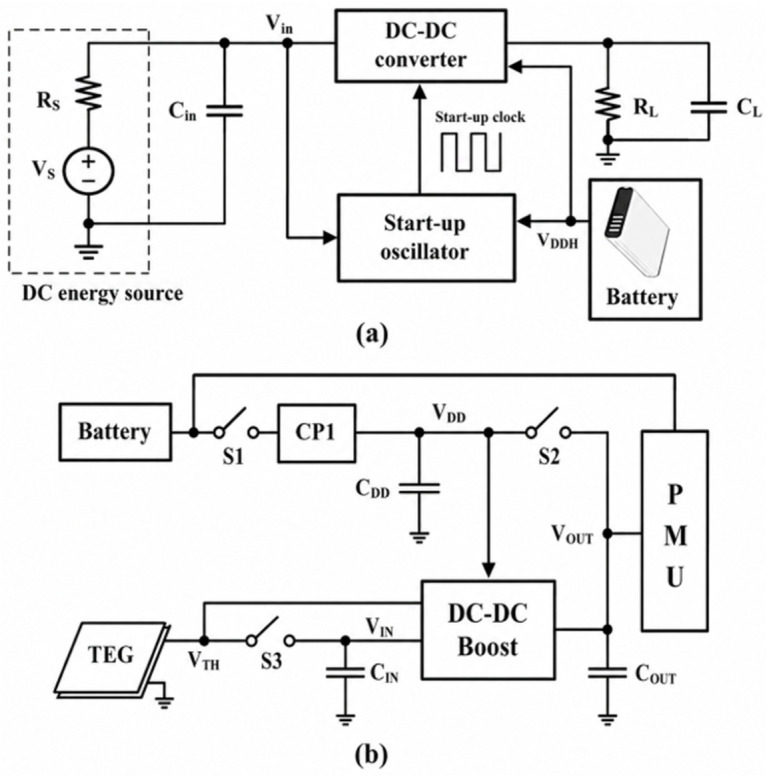
(**a**,**b**) Energy harvesting systems with battery assistance for the start-up [74,75].

Representative system architectures for battery-assisted start-up are illustrated in Figure 4a,b [74,75]. In Figure 4a, the battery provides a high supply rail (*V*_DDH_) that directly powers both the start-up oscillator and the main converter control circuitry, ensuring reliable initialization even in the absence of sufficient EH. In Figure 4b, the battery biases a charge pump or auxiliary block to enable low-voltage operation while also supplying the PMU. Both configurations overcome the initial energy barrier, but they differ in how the auxiliary supply is integrated into the control and start-up path.

To mitigate these limitations, battery-assisted EH has been explored as a hybrid approach [77,78], where the energy harvester extends the service life of a rechargeable battery [79,80]. While this improves lifetime relative to purely battery-powered systems, it does not resolve the fundamental dependence on a component with a finite cycle life. The eventual need for replacement means such systems fall short of the goal of a truly maintenance-free platform.

### 2.2. Mechanical Switch-Assisted Start-Up

**Figure 5:**
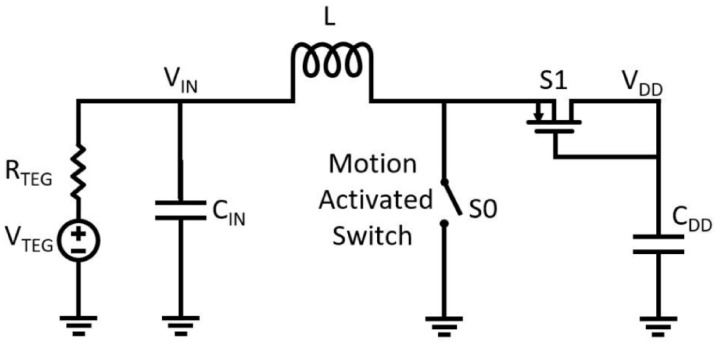
Start-up circuit with mechanical switch assistance [66].

A distinct strategy, especially relevant to wearable applications, leverages kinetic energy from ambient body motion to actuate a mechanical switch [81,82]. As illustrated in Figure 5, vibrations from activities such as walking can trigger a mechanical switch (S_0_) (reported acceleration thresholds can be <100 mg), initiating a multi-step energy accumulation process. The operating principle follows a two-phase cycle: first, closure of S_0_ enables the TEG voltage to build current in an inductor; subsequently, opening of the switch forces this inductor current to charge a capacitor through an ideal diode switch (S_1_). Once the capacitor reaches sufficient voltage, it triggers a transient series RLC energy transfer that delivers the initial pulse required to bootstrap the main converter.

The main advantage of this method is that it achieves a remarkably low Vstart of ≈35 mV with a relatively simple circuit [66]. However, it imposes important practical constraints: it often requires a large inductance and a small capacitance, leading to bulky passive components and a limited power delivery capability. More critically, system operation depends on intermittent and unpredictable motion [83], making start-up timing variable and energy transfer potentially inefficient. These limitations restrict suitability for applications requiring reliable and repeatable cold-start behavior.

### 2.3. Multi-Source Energy Harvesting

A prominent strategy to overcome the cold-start problem is multi-source EH, which uses an auxiliary ambient energy source to bootstrap the system [84,85]. This approach can circumvent the voltage limitations of a single TEG. For instance, some solutions leverage RF energy via an antenna to harvest sufficient power to initiate the main TEG boost converter [9,86]. A more integrated hybrid thermal/RF architecture combines energy from both sources (often via rectification and power-combining) and can achieve operation from TEG inputs on the order of ≈50 mV [87,88]. Other co-design strategies exploit cross-source synergy; for instance, one approach uses the TEG’s DC voltage to bias a device in the RF rectifier path to improve RF-to-DC conversion efficiency [89].

**Figure 6:**
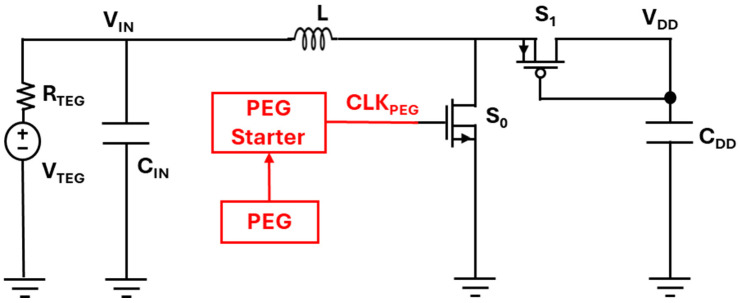
Energy harvesting systems with a PEG start-up [68].

However, even “tens of millivolts” can exceed the ~10 mV available from compact TEGs under minimal ∆T. To bridge this gap, a hybrid TEG and piezoelectric generator (PEG) architecture has been proposed [68], as shown in Figure 6. In this design, the PEG harvests kinetic energy to generate a start-up clock signal (CLK__PEG_), which drives the boost converter so that thermal energy can be accumulated on a storage capacitor even when *V*_IN_ is extremely small.

A fundamental limitation shared by all multi-source approaches is increased system complexity, cost, and form factor. Additional transducers (antennas, piezoelectric elements), dedicated harvesting circuits, and sometimes off-chip components can reduce their suitability for miniaturized, single-primary-source applications.

### 2.4. Transformer-Based Techniques

Transformer-based start-up circuits form a distinct category that leverages the voltage gain of magnetically coupled inductors to achieve exceptionally low start-up voltages [45,90]. This makes them attractive in industrial applications where minimizing required input voltage can take precedence over miniaturization. Off-chip transformer implementations have demonstrated start-up from inputs as low as ≈20–30 [91,92,93], with the threshold often tunable via transformer turns ratio. Enhanced versions that incorporate voltage monitoring can further optimize and handover behavior.

**Figure 7:**
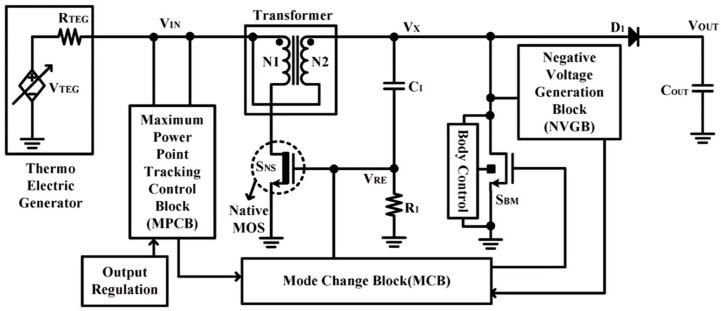
Energy harvesting systems with transformer-based start-up [67].

Specific implementations highlight inherent design compromises. For instance, one design achieves start-up from ≈40 mV and reuses a transformer winding as the main converter’s inductor, improving component utilization [67] as shown in Figure 7. However, such architecture can suffer from low conversion efficiency at *V*_IN_ < 100 mV and introduce integration penalties.

**Figure 8:**
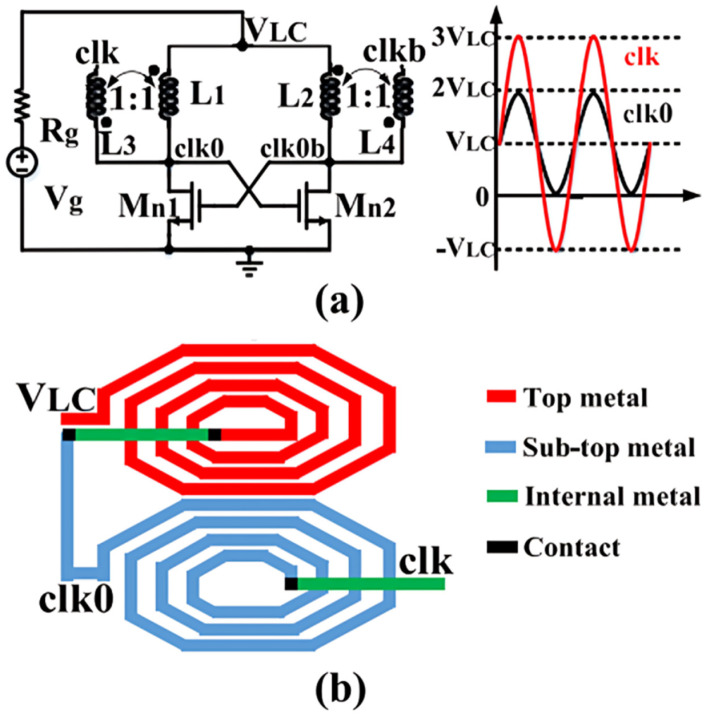
(**a**) Energy harvesting system with a 1:1 LC oscillator start-up. (**b**) On-chip transformer [96].

A key limitation of high-performance transformer-based solutions is reliance on large off-chip transformers, which prevents adoption in highly integrated systems [94,95]. As illustrated in Figure 8, on-chip transformers have been explored to improve integration; however, their performance is constrained by a low-quality factor (Q). For example, stacked-type transformer-based LC oscillators have reported start-up voltages around 100–160 mV and relatively low efficiencies [96]. Similarly, fully integrated voltage multipliers using on-chip transformers for passive clock boosting have reported start-up requirements on the order of ≈85 mV [97].

Despite the low *V*_start_ capability of off-chip transformers, their dependency on external magnetics and the performance penalties of fully integrated transformer variants render this category less practical for compact TEG applications. This is particularly relevant because compact TEGs often generate only ≈40–60 mV under a small ∆T of 1–2K [98,99], a regime where integrated transformer-based solutions struggle to achieve competitive efficiency.

### 2.5. Oscillator-Based Techniques

Oscillator-based start-up circuits represent the final category in this review and are widely regarded as a promising path for ultra-low-voltage TEG EH due to their compatibility with standard CMOS processes and potential for monolithic integration. The core principle is to convert the low TEG DC voltage into an AC signal using an oscillator, then rectify and multiply it (e.g., via charge pump) to generate a voltage sufficient to bootstrap the main PMU [100,101,102]. Oscillators used in such systems are commonly grouped into resonant oscillators or waveform oscillators, each with distinct characteristics [103].

Resonant oscillators (e.g., LC-tank configurations) can offer a strong start-up performance. Reported examples include start-up from ≈50 mV using an LC oscillator with a voltage multiplier [104,105] and cold-start capability range of ≈60–200 mV using a Hartley oscillator that shares an inductor with the boost converter to reduce component count [106]. The most aggressive demonstrations report operation across a range of approximately 11–50 mV using enhanced-swing oscillator approaches with specialized devices and an off-chip inductor [107,108,109]. However, these approaches fundamentally depend on inductive components. While off-chip inductors can enable superior performance, they compromise integration; conversely, on-chip inductors suffer from low Q and large area values, limiting efficiency and practicality.

Waveform oscillators, particularly ring oscillators (ROs), present a more integration-friendly alternative due to their compact, transistor-only implementation, making them attractive for IoT nodes with strict area and cost constraints [64,110]. Conventional RO designs typically require several-hundred millivolts for robust switching, far exceeding the tens of millivolts available from TEGs under small ∆T [111,112]. This limitation has motivated gain-enhancement strategies (specialized delay cell, adaptive biasing, body-bias techniques) to maintain oscillation stability at ultra-low supply voltages.

Overall, oscillator-based start-up circuits establish a clear trajectory: resonant oscillators can achieve very low *V*_start_ but face a fundamental integration barrier due to inductors, whereas ROs offer superior scalability and miniaturization at the cost of challenging millivolt-level design. Ongoing research continues to address these challenges through advanced delay cell architectures and biasing schemes, positioning ROs as one of the most practical solutions for next-generation autonomous systems.

#### 2.5.1. Conventional Ring Oscillator

ROs have emerged as a leading option for integrated start-up clock generation primarily because they can be implemented using transistors alone, enabling compact area and low cost [73,113,114]. Unlike resonant oscillators that require inductive components, ROs are fully CMOS compatible and therefore well suited to miniaturized applications [115].

**Figure 9:**
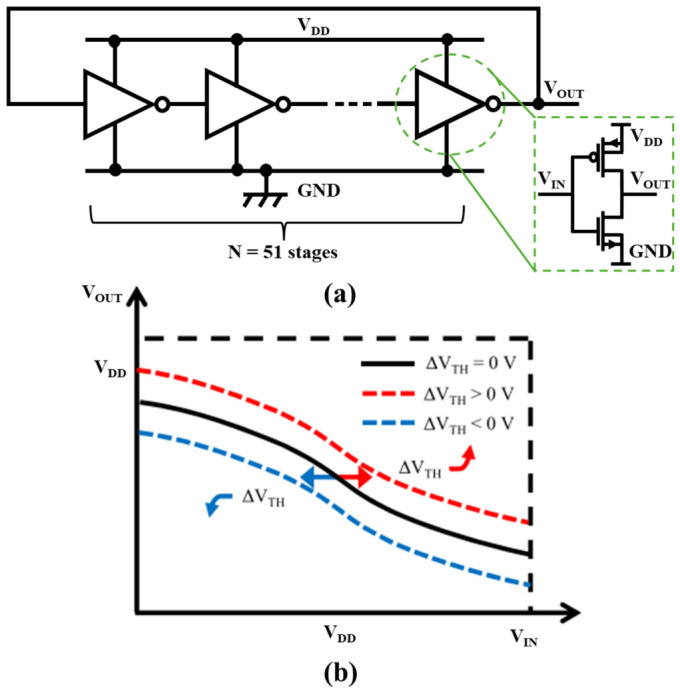
(**a**) Conventional ring oscillator and (**b**) the voltage transfer curve (VTC) with ∆*V*_TH_ as a parameter [116].

A conventional RO, shown in Figure 9a, consists of an odd number of inverter stages connected in a closed loop. Its oscillation frequency is determined directly by the number of stages and propagation delay, enabling straightforward design control [116]. Sustained oscillation requires the magnitude of inverter voltage gain to satisfy:

(5) $$\left|A_{INV}\right|\geq 1$$

Figure 9b illustrates a voltage transfer characteristic (VTC) under ultra-low supply voltages, *V*_DD_ < *V*_TH_, where both transistors operate in a subthreshold regime. The drain current can be approximated as

(6) $$I_{D}=I_{0}exp(\frac{V_{GS}-V_{TH}}{\eta V_{T}})[1-exp(-\frac{V_{DS}}{V_{T}})]$$

where $I_{0}=\mu C_{OX}\frac{W}{L}V_{T}^{2}$ represents the pre-exponential factor; $V_{T}=\frac{k_{B}T}{q}$ is the thermal voltage. The inverter’s switching voltage is derived from current matching *I*_N_ = *I*_P_, yielding:

(7) $$V_{IN}=\frac{V_{DD}+V_{TH,N}+V_{TH,P}}{2}+\frac{\eta V_{T}}{2}ln(\frac{I_{0P}}{I_{0N}}\frac{1-exp(-V_{OUT/V_{T}})}{1-exp(-V_{DD}-V_{OUT})/V_{T}})$$

Differentiating Equation (3) provides the inverter voltage gain $A_{INV}=\frac{{-2exp(-V_{OUT}/V_{T})[1-exp(-\frac{V_{DD}-V_{OUT}}{V_{T}})]}^{2}}{\eta V_{T}[1-exp(-\frac{V_{DD}}{V_{T}})]}$. The maximum gain occurs at *V*_OUT_ = *V*_DD_/2:

(8) $$\left|A_{INV,MAX}\right|=\frac{V_{DD}}{{\eta V}_{T}}exp(-\frac{V_{DD}}{{2V}_{T}}).$$

As Equation (8) clearly demonstrates, the maximum achievable gain decreases exponentially with reducing supply voltage, revealing the fundamental challenge of implementing ROs in ultra-low-voltage EH systems. Equation (7) indicates significant sensitivity to threshold voltage mismatch ∆*V*_TH_ = |*V*_TH,N_ − *V*_TH,P_|, which shifts the VTC horizontally and degrades the effective gain.

As the supply decreases into deep subthreshold, the available inverter gain decreases rapidly, making the loop-gain criterion difficult to satisfy and limiting oscillation amplitude and frequency. Conventional gain-compensation strategies (more stages, wider devices) increase leakage and static power and enlarge area, undermining RO advantages [117,118]. Therefore, the RO-based start-up circuit must balance minimum operating voltage, sufficient oscillation swing, and acceptable overhead under process/voltage/temperature variation.

#### 2.5.2. Ring Oscillator Using Self-Biased Inverter

Conventional CMOS inverters typically require supply voltages far above those available from miniature TEGs. Operating standard inverters at ultra-low voltages degrades the on/off ratio and stage gain, compromising oscillation reliability [119].

**Figure 10:**
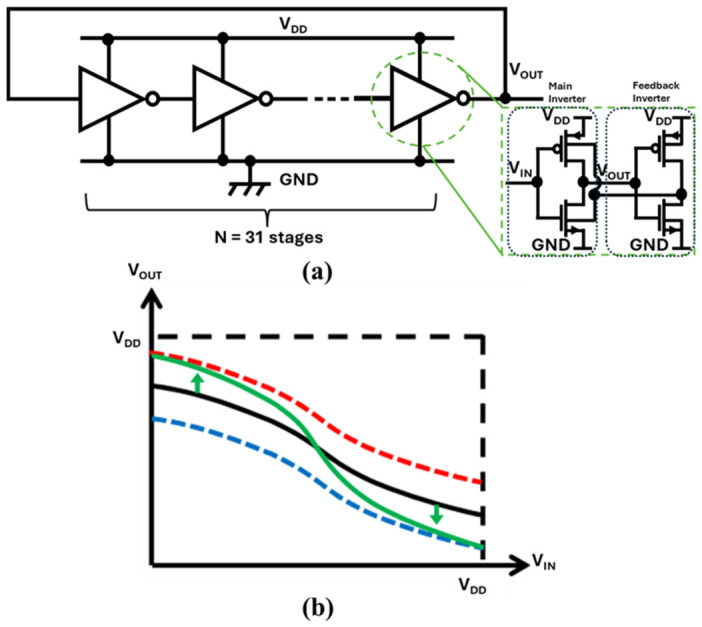
(**a**) Ring oscillator using self-biased feedback inverter and (**b**) voltage transfer curve [116].

To address this limitation, a self-biased inverter (SBI) delay cell has been proposed as a gain-enhanced stage [116]. As illustrated in Figure 10a, the SBI architecture introduces feedback that dynamically adjusts the body bias, effectively modulating threshold voltage (*V*_TH_):

(9) $$V_{TH}=V_{TH0}+\gamma (\sqrt{2\left|\phi_{F}\right|-V_{BS}}-\sqrt{2\left|\phi_{F}\right|}),$$

where *V*_BS_ is the body-source voltage, *V*_TH0_ is the threshold voltage at *V*_BS_ = 0 V, γ is the proportional factor, and ϕ_F_ is the surface potential.

This dynamic biasing sharpens the VTC and increases stage gain, enabling oscillation at significantly lower supply voltages as shown in Figure 10b. SBI-based ROs have reported oscillation down to ≈42 mV, improving suitability for TEG systems [116]. While this reduces the number of stages required compared to conventional inverters, it adds design complexity and can increase overhead due to auxiliary circuitry.

#### 2.5.3. Ring Oscillator Using Stacked Three-Inverter Delay Cell

**Figure 11:**
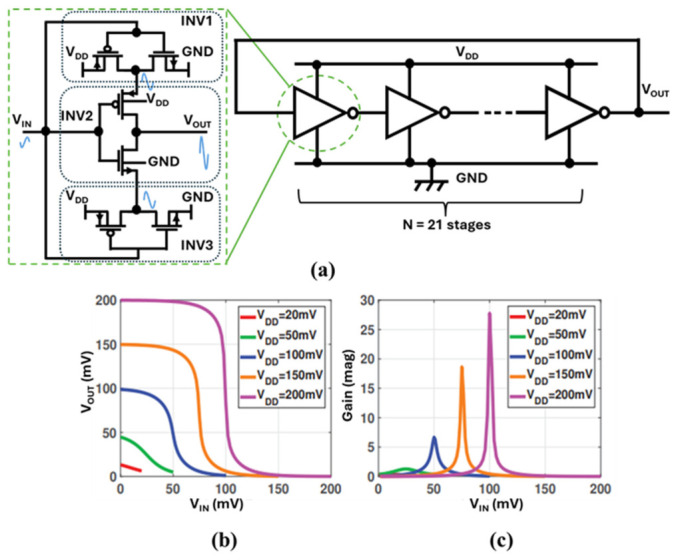
(**a**) Ring oscillator using stacked three inverters, (**b**) voltage transfer curve, and (**c**) DC gain [112].

At very low supplies, RO performance is limited by degraded inverter gain and reduced output voltage swing [119]. To mitigate these limitations, a stacked three-inverter delay cell has been proposed, demonstrating self-sustained oscillation from ≈50 mV [112]. As shown in Figure 11a, the three inverters (INV1, INV2, INV3) are arranged such that the input drives all gates, while a regenerative path improves effective switching behavior and supports higher swing. Proper DC operating point is maintained by device sizing (e.g., sizing the outer device larger than the inner devices) [120].

Figure 11b illustrates inverter VTC degradation as *V*_DD_ decreases; the transition region flattens as gain falls. This behavior is consistent with the widely cited discussion of the “thermal limit” discussion: CMOS inverter switching under ideal subthreshold assumptions at 300 K is often associated with a characteristic voltage scale near ≈36 mV [121]. Importantly, this should be interpreted as a practical lower bound under idealized assumptions, not a strict impossibility, and real circuits may deviate depending on device selection, leakage, and operating conditions [122].

Fabricated in 180 nm CMOS, a 21-stage RO using this cell reported kHz-level oscillation with high swing at tens of millivolts supply [112]. The trade-off is increased area and power due to the higher transistor count and routing complexity per stage. Nonetheless, this approach is valuable where the minimum operating voltage is the dominant constraint.

#### 2.5.4. Ring Oscillator Using Tri-State Buffer

**Figure 12:**
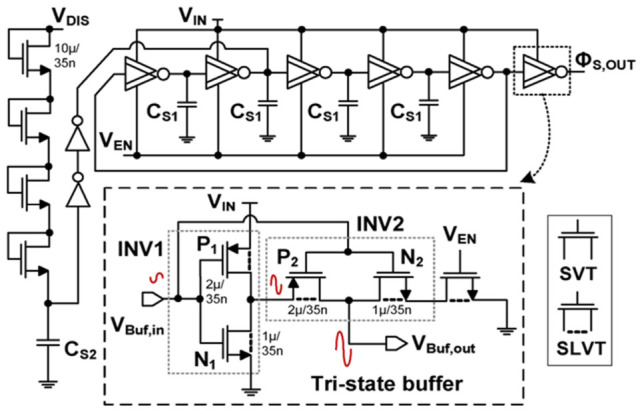
Ring oscillator using tri-state buffer [123].

At TEG-level supplies, conventional ROs are limited by reduced stage gain and reduced output swing, which can impair the ability to drive subsequent stages [119]. A gain-enhanced RO architecture incorporating a tri-state buffer has been introduced to improve effective transconductance and stage gain [123]. As shown in Figure 12, a primary inverter (INV1) drives a tri-state buffer stage (INV2). Use of super low-threshold voltage (SLVT) devices can further lower operating voltage, while tri-state behavior improves signal shaping and propagation.

A common analytical view expresses the inverter gain as:

(10) $$AV1≅\frac{gm,eff1}{gds,N1+gds,P1}=\frac{gm,N1+gm,P1}{gds,N1+gds,P1},$$

where the effective transconductance *g*_m,eff1_ is the transconductance sum of N_1_ and P_1_. The *g*_ds_, N_1_, and g_ds_, P_1_ are the output conductance of N_1_ and P_1_, respectively. The effective transconductance enhancement in the tri-state buffer can be approximated by:

(11) $$gm,\mathrm{eff}2≅\left(1+AV1\right)gm,P2+gm,N2$$

where *g*_m,P2_ and *g*_m,N2_ are the transconductance of P_2_ and N_2_, respectively. The resulting stage gain is:

(12) $$AV2≅\frac{gm,eff2}{gds,P2+gds,N2}=\frac{\left(1+AV1\right)gm,P2+gm,N2}{gds,P2+gds,N2}$$

where *g*_ds,N2_ and *g*_ds,P2_ are the output conductance of N_2_ and P_2_, respectively.

This architecture can provide stronger gain and output swing at low supply, but at the expense of increased transistor count and potential overhead due to device choices and additional circuitry. It is most suitable for scenarios where guaranteed cold-start performance justifies higher resource utilization.

**Figure 13:**
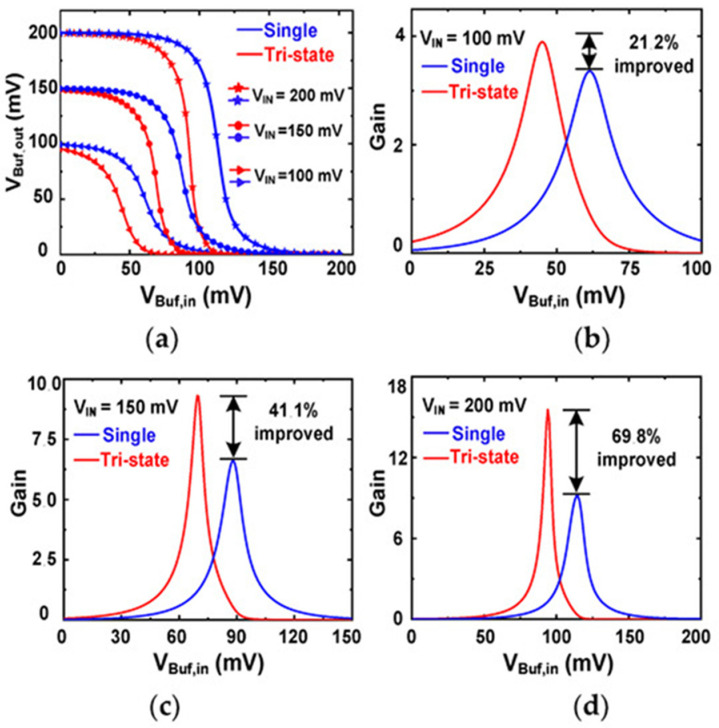
(**a**)**.** Transfer characteristics; gain comparison at (**b**) *V*_IN_ = 100 mV, (**c**) *V*_IN_ = 150 mV, and (**d**) *V*_IN_ = 200 mV [123].

Figure 13a compares the voltage transfer characteristics of a single-stage buffer and tri-state buffers at three *V*_IN_ values. The tri-state buffer exhibits steeper slopes, indicating higher gain. The gain enhancement is further quantified in Figure 13b–d. At *V*_IN_ = 100 mV, a gain improvement of approximately 21.2% has been reported, increasing to 41.1% at 150 mV and 69.8% at 200 mV [123]. These results support the tri-state buffer as an effective gain-enhancement approach for ultra-low-voltage start-up circuits.

## 3. Performance Comparison

Building on the system-level motivation introduced in Section 1 and the taxonomy of start-up techniques reviewed in Section 2, this section consolidates the taxonomy introduced in Section 2 and provides a comparative interpretation across three levels: (a) category-level trade-offs (Table 1), (b) topology-level trade-offs within RO start-up circuits (Table 2), and (c) benchmarking of representative reported implementations (Table 3).

### 3.1. Cross-Category Comparison of Start-Up Strategies (Table 1)

Table 1 compares five major classes of start-up techniques for ultra-low-voltage TEG-EH systems, highlighting their implications for start-up voltage, integration level, autonomy, and cost. From an energy-conversion perspective, the key observation is that minimizing start-up voltage alone is insufficient; practical deployment additionally requires scalability, long-term autonomy, and compatibility with compact system architectures.

Battery-assisted start-up schemes provide highly reliable initialization by externally supplying the required control bias. However, they fundamentally undermine the objective of energy autonomy and introduce maintenance, replacement, and environmental concerns, which are incompatible with long-term, maintenance-free operation. Mechanical switch-assisted techniques can achieve very low apparent start-up voltages by externally triggering energy transfer, but their dependence on intermittent mechanical excitation and bulky passive components limits repeatability and robustness, particularly in unattended or implantable systems.

Multi-source energy harvesting approaches reduce the effective cold-start barrier by supplementing thermal energy with auxiliary sources such as RF or kinetic energy. While this improves start-up robustness under weak temperature gradients, it increases system complexity, transducer count, and cost, which can negate the benefits of single-source TEG solutions. Transformer-based start-up architectures achieve some of the lowest reported start-up voltages due to magnetic voltage gain, but their reliance on off-chip magnetics constrains miniaturization and prevents full system-on-chip (SoC) integration.

In contrast, oscillator-based start-up techniques provide the most direct pathway to fully integrated, CMOS-compatible implementations. Although their achievable start-up voltage is typically higher than that of transformer-based or mechanically assisted schemes, they preserve autonomy, reduce form factors, and enable scalable manufacturing. Table 1 therefore reveals a fundamental trade-off: as start-up architectures evolve toward full integration and autonomy, the achievable start-up voltage becomes increasingly governed by circuit-level gain generation and loss mechanisms rather than external voltage amplification.

### 3.2. Comparison of RO Start-Up Topology (Table 2)

Table 2 compares RO delay-cell topologies that enable integrated cold-start operation under deep-subthreshold conditions. Because oscillator-based architecture offers the highest level of CMOS compatibility among start-up techniques, the comparison focuses on how different RO designs address-gain degradation and voltage swing limitations at ultra-low supply voltages.

Conventional inverter-based ROs offer minimal area and design simplicity; however, their insufficient small-signal gain and degraded voltage transfer characteristics severely limit operation below 50 mV. To address this limitation, gain-enhanced RO variants employ architectural techniques that improve effective transconductance at low supply voltages. Self-biased inverter ROs dynamically modulate device thresholds to enhance switching behavior, achieving reduced start-up voltage with moderate overhead. Stacked three-inverter delay cells introduce regenerative feedback paths that support oscillation near the practical low-voltage boundary, albeit at the cost of increased transistor count and routing complexity. Tri-state buffer-based ROs further enhance effective gain through bootstrapping, providing a strong output swing and improved robustness when driving rectification and charge-pump stages, although they typically require additional circuitry and low-threshold devices.

From an energy-conversion perspective, Table 2 highlights that RO-based cold-start operation is fundamentally governed by the trade-off between effective gain generation and parasitic loss under extreme current constraints. Achieving lower start-up voltage generally necessitates increased architectural complexity, underscoring the need for careful co-optimization of area, leakage, and robustness to process, voltage, and temperature (PVT) variation in fully integrated TEG energy harvesting systems.

### 3.3. Benchmarking of Reported Start-Up Implementations (Table 3)

Table 3 benchmarks representative reported start-up implementations across the architectural spectrum. A clear pattern emerges: the lowest start-up voltages are typically achieved using external components such as batteries, mechanical switches, off-chip transformers, or auxiliary harvesters. While effective from a voltage standpoint, these solutions incur penalties in system size, cost, and integration suitability, limiting their relevance for compact TEG-powered devices.

Among fully integrated solutions, a performance gradient is observed. Charge-pump-based start-up circuits typically require higher input voltages but offer simplicity and ease of integration. On-chip transformers or LC-based approaches reduce the start-up threshold but remain constrained by low-quality-factor magnetics and associated efficiency penalties. RO-based start-up circuits occupy the most practical region for monolithic systems, with reported start-up voltages typically in the tens of millivolts, depending on the chosen gain-enhancement strategy and device assumptions. These observations reinforce the conclusion that further reductions in start-up voltage within standard CMOS technologies will primarily rely on architectural innovation rather than incremental topology refinement.

### 3.4. Application Scenarios and Reported TEG Performance

The reviewed start-up circuits target different TEG operating environments, and their practical suitability depends strongly on the available temperature gradient, source impedance, form-factor constraint, and load profile. For wearable and biomedical platforms, the available thermal gradient is often only a few kelvin. Therefore, compact TEGs may produce input voltages in the tens of millivolts and power levels ranging from microwatts to low milliwatts. In this regime, body-heat harvesters and battery less sensor interfaces require cold-start thresholds below a range of approximately 35–70 mV together with very low quiescent control power [32,38,66,73]. For IoT sensor nodes placed near machinery, building surfaces, or industrial waste-heat sources, the thermal gradient can be more stable and higher, allowing start-up voltages near a range of 70–190 mV and enabling higher steady-state output power after the boost converter enters normal operation [32,37,56,57]. In automotive and battery thermal-management applications, the larger heat flux and physical volume can justify transformer-assisted or hybrid approaches because the priority may shift from full monolithic integration to high conversion efficiency and robust cold start [26,79,80].

Ultra-low-power electronic systems, such as wearable health-monitoring sensors, biomedical implants, wireless IoT sensor nodes, industrial condition-monitoring devices, automotive sensors, and battery thermal-management systems, are frequently powered by harvested thermoelectric energy. The target application has a significant impact on PMU design. Wearable and biological systems are typically subjected to modest and unstable temperature gradients, necessitating low start-up voltage, low leakage, compact size, and full integration. IoT sensor nodes frequently function intermittently, necessitating efficient energy storage, load gating, and regulated output delivery for sensing, computing, and wireless communication. Industrial and automotive systems may produce larger thermal gradients, but the PMU must handle larger input voltage variation, higher operating temperatures, and fluctuating heat sources.

From an application point of view, no single start-up circuit is usually ideal. Battery-assisted and mechanically aided circuits are appropriate when consistent triggering is more critical than maintenance-free autonomy. Multi-source circuits are appealing when an additional ambient source, such as vibration, RF, or piezoelectric energy, is readily available. Transformer-based circuits are beneficial when the input voltage is extremely low and off-chip magnetics are permitted. Fully integrated oscillator-based circuits, particularly RO-based variations, are best suited to miniaturized wearable, implantable, and IoT devices where volume, cost, and CMOS compatibility are critical. Therefore, start-up topology selection should not be based only on the minimum start-up voltage. It must also consider output-power demand, storage capacity, load profile, form factor, reliability, and whether off-chip components are acceptable. This application-oriented interpretation complements the topology-oriented comparison in Table 1, Table 2 and Table 3 and clarifies how reported TEG performance should be matched to the intended deployment scenario.

### 3.5. Topology Selection Guide for TEG Start-Up Design

**Figure 14:**
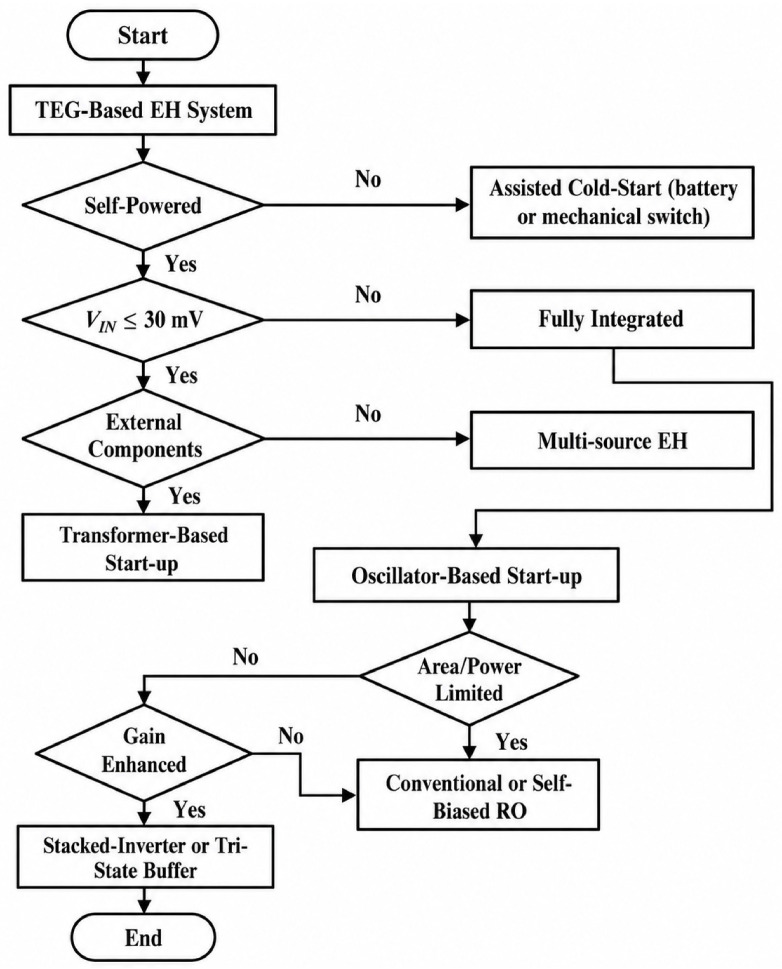
Design-oriented topology selection guide for ultra-low-voltage TEG start-up circuits, illustrating the relationship between input voltage regime, external component dependency, integration level, and suitable architectural choices for autonomous energy harvesting systems.

To consolidate the insights from Table 1, Table 2 and Table 3, Figure 14 presents a topology selection guide for ultra-low-voltage start-up circuits in TEG-EH systems. The guide first distinguishes whether a true self-powered cold-start operation is required. If external assistance is acceptable, battery- or mechanically assisted solutions provide robust initialization but compromise autonomy or determinism. For fully self-powered systems, the selection depends on the available TEG input regime and the permissibility of external passive components. When full integration is required, oscillator-based start-up architectures emerge as the default choice, with RO topology selection guided by acceptable overhead, target start-up voltage, and robustness requirements. This guide is intended to support architecture-level decision-making for energy harvesting systems rather than circuit-level optimization alone.

## 4. Discussion and Future Directions

The design of start-up circuits for TEG-based energy harvesting has reached a point where reducing *V*_start_ further involves clear practical trade-offs. Techniques based on transformers or mechanical switches have demonstrated impressively low start-up voltages, in some cases between 20 and 35 mV. However, these gains are achieved at the cost of bulky off-chip components, which makes such approaches difficult to scale for compact IoT and biomedical applications where size, cost, and integration are critical. For these reasons, RO-based architecture has emerged as a more realistic solution for fully autonomous and highly integrated systems. Their compatibility with standard CMOS processes enables compact, monolithic implementations that are well-suited for large-scale deployment. At the same time, RO-based designs face inherent limitations when operating deep in the subthreshold regime. As the supply voltage approaches the millivolt range, inverter gain and output swing degrade rapidly, reflecting the fundamental constraints of thermal carrier transport often referred to as the Boltzmann limit. This helps explain why reported RO start-up voltages in recent work tend to cluster in the range of 40–60 mV, suggesting that further reductions will be increasingly difficult to achieve through circuit topology alone.

In addition to lowering *V*_start_, ensuring reliable system behavior during the transition from cold start to normal operation remains a major challenge. If the main boost converter is enabled too early, the supply can collapse and force a repeated restart cycle; if enabled too late, valuable harvested energy is wasted. Addressing this balance requires more adaptive handover strategies that respond to the actual energy available in storage elements, rather than relying solely on fixed voltage thresholds. Looking ahead, meaningful progress in TEG-based EH is unlikely to come from isolated circuit optimizations in a single block. Instead, future advances will depend on coordinated design across devices, circuits, and system-level control, with careful attention to process, voltage, and temperature variations. Establishing common figures of merit that capture start-up capability, integration level, efficiency, and robustness would also help provide clearer benchmarks and a guide to the development of truly autonomous EH platforms.

A practical TEG source is rarely static. Because the temperature gradient, thermal contact resistance, heat-flow channel, and load state might change dynamically over time, the generated voltage and power fluctuate. For wearable TEGs, body motion and ambient airflow can alter the thermal gradient, whereas in industrial or automotive environments, waste heat may fluctuate with operating cycles. As a result, TEG power-management circuits must support time-adaptive operation. Adaptive techniques include dynamic maximum power point tracking, switching frequency adjustment, pulse-frequency modulation under light-load conditions, zero-current or zero-voltage switching control, adaptive undervoltage lockout, and controlled load connection only when sufficient stored energy is available. Time adaptivity is especially critical in cold-start circuits when transitioning from the start-up block to the main boost converter. If the main converter is turned on too soon, the internal supply may fail; if it is turned on too late, the captured energy is lost. As a result, future TEG PMUs should integrate low-voltage start-up capability with adaptive control that reacts to the instantaneous energy available from the TEG and storage element.

In addition to start-up voltage, the energy overhead of the power-management circuit is an important design consideration. Several PMU sub-blocks, including the oscillator, voltage detector, MPPT controller, gate drivers, reference generator, protection circuit, and output regulator, use energy during start-up and steady-state operations. For ultra-low-voltage TEG systems, the harvested power under tiny temperature gradients may be severely limited; thus, the PMU must consume significantly less power than the gathered source. Otherwise, a circuit may display cold-start capability while providing little or no net energy to the load. This trade-off is especially critical in oscillator-based start-up circuits, where decreasing the start-up voltage frequently necessitates additional gain-enhancement circuitry, body-biasing networks, or charge-pump stages. These blocks improve cold-start performance while potentially increasing leakage, area, and dynamic power. Thus, future comparisons should consider not only V__start_ and peak efficiency, but also start-up energy, quiescent current, control-circuit overhead, and the ratio of delivered load energy to harvested TEG energy.

## 5. Conclusions

This paper presented a design-oriented architectural review of start-up circuit techniques addressing the cold-start challenge in thermoelectric EH systems. Five major categories, external energy assistance, mechanical switch-assisted, multi-source harvesting, transformer-based, and oscillator-based architectures, were analyzed in terms of operating principles, integration feasibility, and practical limitations. The comparative analysis highlights a fundamental trade-off between achieving ultra-low start-up voltage and maintaining system autonomy, scalability, and monolithic integration. While external and passive-component-based approaches can demonstrate exceptionally low start-up voltages, they inherently compromise integration and long-term autonomy. In contrast, oscillator-based solutions, particularly advanced RO architectures employing gain-enhancement techniques, offer the most balanced pathway toward fully integrated, autonomous TEG-powered systems. Future progress is expected to rely on coordinated device-circuit-system co-design and standardized evaluation methodologies, rather than isolated reduction in start-up voltage alone.

## Figures and Tables

**Table 1:** Comparison of start-up strategies for ultra-low-voltage thermoelectric EH systems.

Category	Start-Up Voltage ^1^	Efficiency	Cost	Integration Level	Component Requirements	Autonomy	Key Limitations	Ref.
**External Battery**	N/A (externally biased)	High (post start-up)	High	Low	Battery or pre-charged storage	No (maintenance dependent)	Breaks energy autonomy; finite lifetime; replacement required	[74,75,76]
**Mechanical Switch**	~35 mV	Low-Moderate	Low-Moderate	Low	MEMS/mechanical switch, large L/C	Conditional (motion-dependent)	Unpredictable operation; bulky passives; poor repeatability	[81,82]
**Multi-Source EH**	~10–50 mV (source-dependent)	Moderate	High	Medium	Additional transducers (RF, piezo, solar)	Conditional (auxiliary source required)	Increased complexity, area, and cost	[84,85,86,87,88,89]
**Transformer-Based**	~20–40 mV	Low-Moderate	High	Low-Medium	Off-chip magnetic components	Yes (post start-up)	Bulky magnetics, poor scalability, and limited integration	[90,91,92,93]
**Oscillator-Based**	~40–100 mV (lower with enhancement)	Moderate	Low	High	Fully CMOS (optionally off-chip passives)	Full autonomy	Gain degradation at ultra-low voltage; design complexity	[100,101,102,103,104,105,106,107,108,109,110,111,112,113,114,115,116,117,118,119,120,121,122,123]

^1^ Start-up voltage refers to the minimum input voltage at which the system can autonomously transition from cold start to regulated operation.

**Table 2:** Comparison of RO delay-cell topologies for integrated ultra-low-voltage start-up.

Metric	Conventional RO	Self-Biased Inverter RO	Stacked Three-Inverter RO	Tri-State Buffer RO
**Start-up Voltage**	100–200 mV	~40–60 mV	~40–50 mV	~50–60 mV
**Gain & Output Swing at low V_DD_**	Poor	Moderate-High	High	Very High
**Power Consumption**	Low	Moderate	Moderate-High	Moderate
**Area & Transistor Count**	Very Low	Moderate	High	Moderate
**Process Scaling Behavior**	Favorable	Favorable	Moderate	Moderate
**Sensitivity to Mismatch & Temp**	High	Moderate	Moderate-High	Low-Moderate
**Integration Simplicity**	Excellent	Good	Moderate	Moderate

**Table 3:** Benchmarking of representative ultra-low-voltage start-up implementations for TEG-EH.

Technique	Architecture Class	Reported Start-Up Voltage (mV)	CMOS Process	Reported Core Area * (mm^2^)	External Components	Key Observation	Ref.
**Battery-assisted PMU**	External assist	300/100	130 nm	~1.16	Battery	Reliable initialization; non-autonomous	[65]
**Mechanical Switch**	Passive assist	~35	350 nm	N/A	switch, L/C	Very low V_start_; event-driven operation	[66]
**RF-assisted TEG**	Multi-Source EH	~50	130 nm	N/A	Antenna	Dependent on RF availability	[86]
**PEG-assisted TEG**	Multi-Source EH	~10–20	180 nm	~0.4	Piezo element	Lowest effective V_start_; bulky system	[68]
**Off-chip Transformer**	Transformer-based	~20–30	130 nm	~0.09	Transformer	Excellent V_start_; limited integration	[45]
**On-chip LC Oscillator**	Transformer-based	~85–160	180 nm	~0.35	None	Efficiency limited by low-Q magnetics	[96]
**Charge-pump start-up**	Fully integrated	~100–200	28 nm	~0.0363	None	Simple integration; higher V_start_	[32]
**RO (Self-Biased)**	Oscillator-based	~42	180 nm	~0.0153	None	Balanced V_start_ and area	[115]
**RO (Stacked Inverter)**	Oscillator-based	~40–50	180 nm	~0.0025	None	Operates near inverter voltage limit	[112]
**RO (Tri-state Buffer)**	Oscillator-based	~50–90	28 nm	~0.003	None	High gain; increased overhead	[123]

* Reported areas and start-up voltages are taken from respective publications and may not be directly comparable due to differences in process node, operating conditions, and measurement methodology.

## Data Availability

Data will be made available on request.

## Abbreviations

The following abbreviations are used in this manuscript:

TEG	Thermoelectric generator
EH	Energy harvesting
RO	Ring oscillator
RF	Radio frequency
V_IN_	Input voltage
V_OUT_	Output voltage
V_start_	Start-up voltage
V_DDH_	High supply rail
V_DD_	Supply rail
PMU	Power management unit
PEG	Piezoelectric generator
CLK__PEG_	Start-up clock signal
VTC	Voltage transfer characteristic
A_INV_	Inverter voltage gain
SBI	Self-biased inverter
V_OC_	Open circuit voltage
